# High-efficiency, polarization-independent broadband metasurface absorber with angular stability for ISM applications

**DOI:** 10.1371/journal.pone.0339385

**Published:** 2026-03-19

**Authors:** Abdulrahman Ahmed Ghaleb Amer, Zaid Ahmed Shamsan, Abdullah S. Algamili

**Affiliations:** 1 Institute for Mathematical Research (INSPEM), Universiti Putra Malaysia (UPM), Serdang, Selangor, Malaysia; 2 Faculty of Engineering, Department of Electrical Engineering, Sana’a University, Sana’a, Yemen; 3 Department of Electrical Engineering, College of Engineering, Imam Mohammad Ibn Saud Islamic University (IMSIU), Riyadh, Saudi Arabia; 4 School of Electrical and Electronic Engineering, Universiti Sains Malaysia, Engineering Campus, Nibong Tebal, Penang, Malaysia; Galgotias College of Engineering and Technology, Greater Noida, INDIA

## Abstract

This study presents a high-efficiency broadband metasurface (MS) absorber designed for applications in the Industrial, Scientific, and Medical (ISM) frequency bands, specifically at 2.4 GHz and 5.8 GHz. The proposed absorber offers wide-angle stability and polarization insensitivity within a compact structure. It consists of a split-ring resonator (SRR) integrated with lumped resistors, hosted on an FR4 substrate and backed by a copper ground plane. An optimized 8 mm air gap is introduced to improve impedance matching and extend the absorption bandwidth without increasing the overall profile. The absorber’s performance was evaluated through theoretical analysis, full-wave electromagnetic simulations, and experimental measurements. The simulated results show near-unity absorption, exceeding 98% at 2.4 GHz and 5.8 GHz under normal incidence. Additionally, the absorber achieves broadband absorption spanning 2.1 to 6.96 GHz for both transverse electric (TE) and transverse magnetic (TM) polarizations, maintaining stable operation for incidence angles up to 60°. The measured results closely agree with simulations, confirming the reliability of the design. Compared with previously reported absorbers, the proposed structure demonstrates enhanced bandwidth, compactness, and angular stability, making it a strong candidate for wireless communication, electromagnetic interference (EMI) shielding, and sensing applications.

## 1. Introduction

Metamaterials are artificially engineered composite structures that exhibit electromagnetic properties not typically found in natural materials. By carefully designing their unit-cell geometry and arrangement, metamaterials can manipulate the fundamental characteristics of electromagnetic waves, including phase, amplitude, and polarization. These exceptional properties arise from their subwavelength periodic structure and the distinct materials from which they are composed. Beyond their unusual electromagnetic behavior, researchers have increasingly explored their resonant characteristics for developing compact, high-performance devices for various applications [1–3]. Due to these unique properties, metamaterials have recently attracted much attention for use in different applications, including radar cross-section (RCS) [4–6], antennas [7], invisible cloaking [8], perfect absorbers [9–13], energy harvesting [14–17], and sensing applications [18]. Metamaterial absorbers have garnered widespread attention due to their remarkable absorption properties and versatility in various applications, including energy harvesting and compact wireless components. They also demonstrate the potential for achieving high performance in miniaturized configurations [19]. The electromagnetic response of metamaterials, specifically their effective permittivity (ε) and permeability (μ), can be precisely tailored by adjusting the geometry of their electric and magnetic unit elements, enabling impedance matching with free space for near-unity absorption. The concept of a perfect metamaterial absorber was first proposed by Landy et al. in 2008 [20], demonstrating the advantages of compactness and low-profile configuration compared to traditional absorbers. Building on this concept, a metasurface (MS) absorber is an ultra-thin, two-dimensional form of a metamaterial absorber composed of periodic subwavelength resonant elements. By matching its impedance to free space and utilizing a metallic ground plane to suppress transmission, it efficiently converts incident electromagnetic energy into heat. Its planar geometry and design flexibility make it ideal for compact, tunable, and broadband absorption applications.

Metamaterial absorbers often exhibit a narrow bandwidth due to their inherent resonant behavior, which constrains their use in applications that require broader spectral coverage. To address this limitation, several approaches have been proposed to improve the absorption bandwidth, including the use of multilayer [21,22] and multi-resonance structures [23, 24]. However, these approaches typically increase fabrication complexity and may not fully achieve the desired bandwidth enhancement. Other studies have explored advanced materials, such as graphene and silicon [25–27], though both present practical challenges: graphene suffers from intrinsic ohmic losses and high fabrication costs, while silicon-based structures require complex etching and lack mechanical flexibility. Recently, metamaterial absorbers incorporating lumped resistors have been proposed to broaden absorption by introducing controlled ohmic losses that dissipate incident electromagnetic energy as heat [28–32]. The addition of resistive elements improves impedance matching and reduces reflection, thereby increasing absorption efficiency. Furthermore, the resistors lower the resonance Q-factor, effectively broadening the bandwidth while maintaining structural simplicity. However, resistive losses alone are often insufficient to achieve wideband absorption. To address this, recent studies have introduced air gaps within resistance-loaded structures, which further enhance impedance matching and modify the effective permittivity and permeability of the absorber, thereby expanding the operational bandwidth [33–40]. While this approach improves performance, prior designs frequently suffer from bulky configurations, poor angular stability, and fabrication difficulties. For instance, in [35], a metamaterial absorber with four lumped resistors and a 15 mm air layer achieved a wider bandwidth at 2.4 GHz, but the large air gap compromised compactness and angular stability. Similarly, in [36], an absorber incorporating 16 resistors and a 12.5 mm air gap operated at 2.1, 5.1, and 12.8 GHz but required a complex structure with limited angular performance. In [37], a dual-band absorber utilizing four resistors and two FR4 layers separated by a 16 mm air gap operated at 2.45 and 5.8 GHz; however, it exhibited poor angular stability and a narrow bandwidth. Likewise, in [38], a broadband metamaterial absorber comprising four lumped resistors and a 13 mm air spacer operated over 2.11–3.89 GHz, achieving a fractional bandwidth of 59.3%. Furthermore, in [39], a broadband absorber with four resistor loads and a 17.5 mm air spacer achieved a fractional bandwidth of 61.2% across the frequency range of 5.44–10.21 GHz. Overall, these designs demonstrated improved bandwidths but remained bulky and challenging to fabricate, underscoring the need for compact, broadband, and fabrication-friendly absorber architectures.

In light of these challenges, this study presents a broadband, high-efficiency MS absorber optimized for operation at the widely used ISM frequencies of 2.4 GHz and 5.8 GHz. Unlike traditional narrowband or dual-band designs, the proposed absorber achieves broadband absorption with enhanced peak absorptivity, optimized impedance matching, and reduced structural complexity. The structure consists of a plus-shaped split-ring resonator (SRR) integrated with four lumped resistors on an FR4 substrate, backed by a solid copper ground plane. An optimized 8 mm air gap is introduced to maximize absorption bandwidth while maintaining a compact profile. This design achieves a practical balance between angular stability, polarization insensitivity, and ease of fabrication, making it suitable for next-generation wireless communication, sensing, and stealth applications.

The key contributions of this research can be summarized as follows:

1. A novel design and comprehensive analysis of a broadband MS absorber optimized for the ISM bands at 2.4 GHz and 5.8 GHz, with particular emphasis on compactness, fabrication simplicity, and ease of integration into wireless systems.
2. Demonstration of near-unity absorption, exceeding 99% absorptivity at both target frequencies under normal incidence.
3. Realization of a broadband performance across the frequency range of 2.1 GHz to 6.96 GHz, with polarization-insensitive absorption and high stability under oblique incidence, confirming its suitability for practical electromagnetic applications.

A comprehensive electromagnetic analysis, including assessments of electric field and surface current distributions, is conducted to clarify the absorption mechanism. The design has been experimentally validated through a fabricated prototype, with measured results closely aligning with simulation predictions. This proposed approach offers a scalable and cost-effective solution for broadband electromagnetic absorption, demonstrating strong potential for practical use in compact, high-performance RF systems.

## 2. Metasurface design

**Fig 1:**
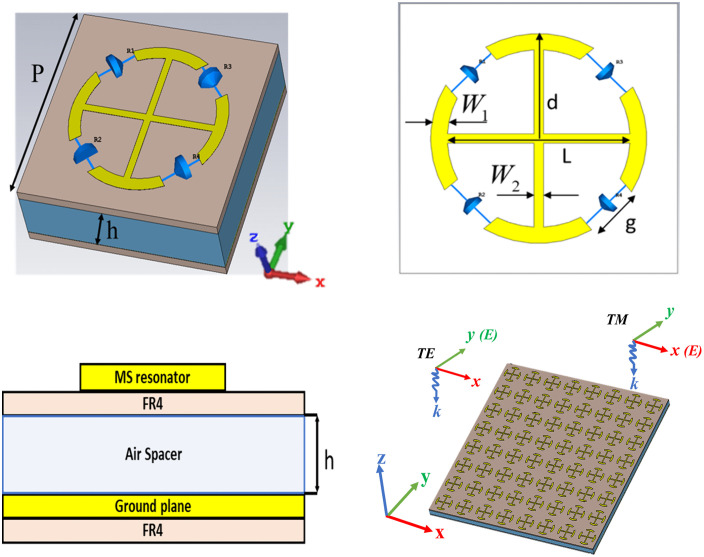
Structural layout of the MS absorber, (a) perspective view, (b) front view, (c) cross-sectional side view, and (d) perspective view of an 8 × 8 array.

The proposed unit cell of the MS absorber, depicted in Fig 1, incorporates a split-ring resonator (SRR) formed by a plus-shaped copper strip, with four 400 Ω lumped resistors placed at its splits. This design is fabricated on a thick FR4 substrate ($\varepsilon_{r}=4.3$, δ=0.0025, and a thickness of 1.6 mm), with optimized dimensions of P = 35 mm, L = 23 mm, d = 6.8 mm, and g = 7. An 8 mm air gap separating the dielectric substrate from the copper ground plane significantly enhances the absorption bandwidth. The electromagnetic energy absorbed by the SRR is efficiently dissipated via the resistors, achieving near-unity absorption. The top SRR and the ground plane are made of copper with a conductivity of 5.8 × 10⁷ S/m and a thickness of 35 μm. Numerical simulations were carried out using the frequency-domain solver in CST Microwave Studio. The structure was precisely modeled by applying periodic boundaries along the x and y axes, while open boundaries were used along the z-axis. Floquet ports were used to excite the structure, allowing for the investigation of both TE and TM polarization modes.

## 3. Results and discussion

**Fig 2:**
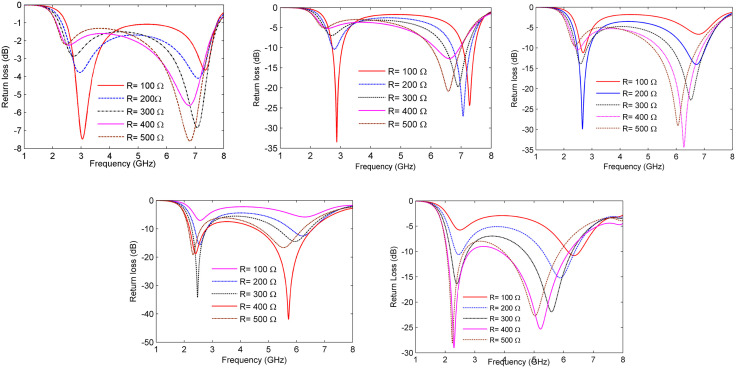
Reflection coefficients under various lumped resistors ranging from 100 Ω to 500 Ω, correspond to air spacer heights of (a) h = 2 mm, (b) h = 4 mm, (c) h = 6 mm, (d) h = 8 mm, and (e) h = 10 mm.


The reflection characteristics of the proposed MS absorber were systematically evaluated by varying the air gap height (h) from 2 mm to 10 mm, alongside adjusting lumped resistor values from 100 Ω to 500 Ω. For each configuration of the air gap, the resistor value was optimized to examine its impact on resonant behavior and absorption efficiency. As illustrated in Fig 2(a–d), an increase in the air gap results in a noticeable downward shift in the resonant frequencies, aligning with the LC resonance relationship described by equation (1), which indicates that the effective capacitance and inductance of the structure change with geometrical spacing.

$$f=\frac{\text{1}}{2\pi \sqrt{LC}}$$ (1)

The findings demonstrate that both the air gap and resistor value are crucial in tuning the spectral response of the absorber. Optimal performance was attained with an air gap of 8 mm and a resistor value of 400 Ω. Under these conditions, the structure exhibits reflection coefficients of −18 dB at 2.4 GHz and of −40 dB at 5.8 GHz, as shown in Fig 2(d). These values correspond to absorptivity levels exceeding 98% at both frequencies, confirming the effectiveness of the chosen design parameters. The pronounced dips in reflection indicate strong impedance matching at both resonant frequencies, validating the broadband operation and the efficacy of resistive loading for enhanced absorption. This outcome substantiates the air gap and resistor configuration derived from the parametric analysis and endorses their selection in the final unit cell design. Additionally, it highlights the tunability of the structure, allowing it to be adaptable for other frequency bands through straightforward geometric and resistive modifications.

### 3.1. Absorption of the MS absorber

The absorptivity, A(ω), is defined based on the S-parameters as follows


$$A(\omega )=1-|S_{11}(\omega ){|}^{\text{2}}-|S_{21}(\omega ){|}^{\text{2}}$$ (2)

where $S_{11}\left(\omega \right)$ is the reflectance and $S_{21}\left(\omega \right)$ is the transmittance. To achieve near-unity absorption, it is essential to minimize both reflectance and transmittance at the target frequency band. The solid copper ground plane plays a crucial role in blocking electromagnetic wave transmission, effectively reducing transmittance through the structure to near zero. Therefore, the absorptivity can be described as


$$A(\omega )=1-|S_{11}(\omega ){|}^{\text{2}}$$ (3)

**Fig 3:**
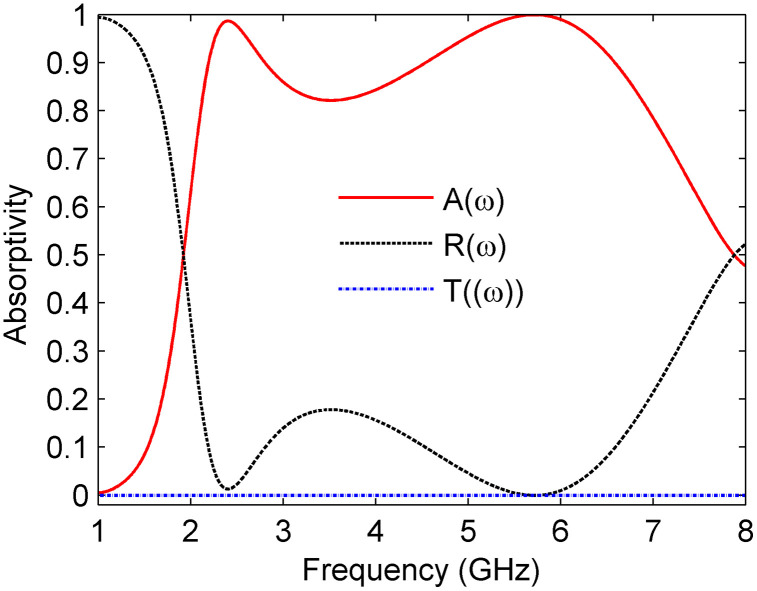
Absorptivity, reflectance, and transmittance under normal incidence.

Fig 3 illustrates the simulated results for absorptivity and reflectance of the designed MS absorber under normal incidence. It can be observed that the reflectance is minimal at frequency bands of 2.4 GHz and 5.8 GHz. Moreover, near-unity absorptivity exceeding 99% is achieved at both frequencies. Furthermore, a wide fractional bandwidth of approximately 107% is achieved over the frequency range of 2.1–6.96 GHz, confirming the design’s broadband performance.

### 3.2. Evaluation of the design concept

**Fig 4:**
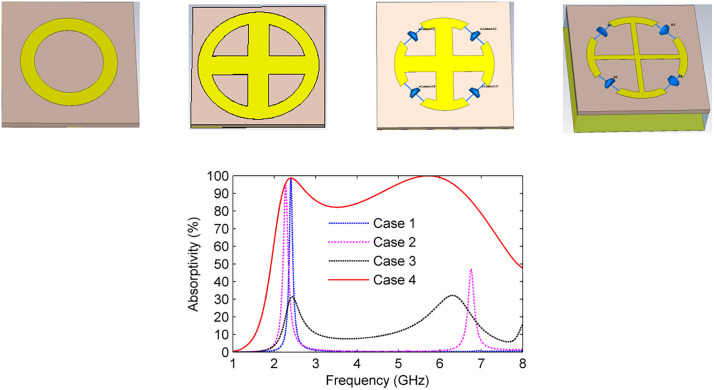
(a) Evolution of the MS absorber design through four design configurations, and (b) Simulated absorptivity characteristics corresponding to each configuration.

The design methodology for the proposed MS absorber is structured into four distinct configurations, as illustrated in Fig 4.

Case 1: A circular resonator is designed on an FR4 substrate and integrated with a solid ground plane, as shown in Fig 4(a). The corresponding absorptivity response in Fig 4(b) demonstrates a peak exceeding 98% at 2.4 GHz. The resonance at this frequency is primarily governed by the dielectric losses of the substrate, resulting in a relatively narrow operational bandwidth.

Case 2: A plus-shaped metallic strip is incorporated into the circular resonator to enhance absorption and introduce multiple resonant modes, as shown in Fig 4(a), Case 2. This modification achieves absorptivity above 90% at 2.4 GHz and approximately 47% at around 6.7 GHz; however, the overall bandwidth remains narrow, as illustrated in Fig 4(b). The primary objective of this configuration is to enable multi-resonant behavior as a foundation for broadband operation.

Case 3: The resonator is further modified by integrating four discrete resistors within its gap regions, as shown in Fig 4(a), Case 3. These resistive elements introduce additional losses that broaden the absorption spectrum, as depicted in Fig 4(b). However, this configuration reduces the peak absorptivity to approximately 30% near both 2.4 GHz and 6.3 GHz due to excessive energy dissipation.

Finally, to further enhance broadband absorption, an 8 mm air gap is introduced between the substrate and the ground plane, as shown in Fig 4(a), Case 4. This adjustment yields nearly perfect absorption exceeding 80% across a continuous frequency range of 2.1 to 6.96 GHz, as shown in Fig 4(b). The bandwidth enhancement is primarily attributed to the air gap, which increases the overall structure thickness, reduces the effective permittivity (ε), and lowers the quality factor (Q), as defined by equation (4).


$$BW=\frac{f_{o}}{Q}=\frac{R}{2\pi L}$$ (4)

where $f_{\text{0}}$ is the operating frequency, Q is the quality factor, R is the resistance, and L is the inductance.

**Fig 5:**
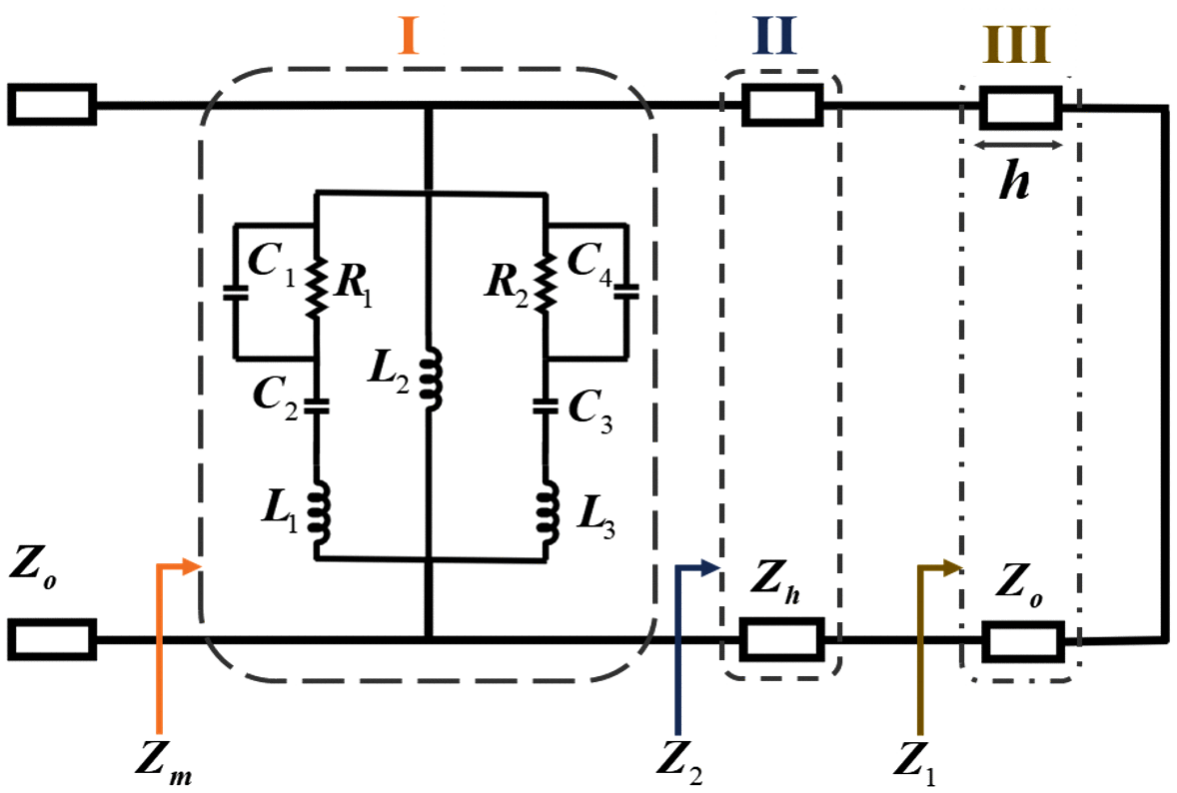
Equivalent circuit. The optimized parameter values are ${\text{C}}_{\text{1}}\text{= 0.155 pF}$, ${\text{C}}_{\text{2}}\text{= 0.11 pF}$, ${\text{L}}_{\text{1}}\text{= 26.4 nH}$, ${\text{L}}_{\text{2}}\text{= 16.35 nH}$, ${\text{L}}_{\text{3}}\text{= 0.7 nH}$, ${\text{C}}_{\text{3}}\text{= 0.1 pF}$, ${\text{C}}_{\text{4}}\text{= 1.05 pF}$ and ${\text{R}}_{\text{1}}{\text{= R}}_{\text{2}}\text{= 400}\Omega$.


The equivalent circuit model of the proposed broadband MS absorber is illustrated in Fig 5 and comprises three primary sections. Section I represents the metallic MS resonator, modeled using parallel inductors, capacitors, and resistors. Section II corresponds to the FR4 substrate, while Section III represents the air gap of height ℎ. Due to the significant thickness of the air gap, electromagnetic coupling between the MS resonator and the ground plane is neglected. In this model, the inductance L characterizes the inductive behavior of the copper structure, whereas the capacitance *C* arises from the gaps in the annular copper resonator. Based on this equivalent circuit representation, the input impedance of the MS configuration is analytically expressed as follows:

$$\begin{array}{c} {\text{Z}}_{A}{\text{\textasciitilde{}=\textasciitilde{}j}\omega \text{L}}_{\text{1}}\text{+}\frac{\text{1}}{{\text{j}\omega \text{C}}_{\text{2}}}{\text{+ (R}}_{\text{1}}\text{||}\frac{\text{1}}{\text{j}\omega \text{C1}}\text{)}\\ {\text{=\textasciitilde{}j}\omega \text{L}}_{\text{1}}\text{+}\frac{\text{1}}{{\text{j}\omega \text{C}}_{\text{2}}}+\frac{R_{\text{1}}}{1+j\omega RC_{\text{1}}} \end{array}$$ (5)

$$Z_{B}=j\omega L_{\text{2}}$$ (6)

$$\begin{array}{c} Z_{C}={\text{j}\omega \text{L}}_{\text{3}}\text{+}\frac{\text{1}}{{\text{j}\omega \text{C}}_{\text{3}}}{\text{+ (R}}_{\text{2}}\text{||}\frac{\text{1}}{{\text{j}\omega \text{C}}_{\text{4}}}\text{)}\\ {\text{=\textasciitilde{}j}\omega \text{L}}_{\text{3}}\text{+}\frac{\text{1}}{{\text{j}\omega \text{C}}_{\text{3}}}+\frac{R_{\text{2}}}{1+j\omega R_{\text{2}}C_{\text{4}}} \end{array}$$ (7)

$$Z_{m}=Z_{A}\left|\right|Z_{B}\left|\right|Z_{C}$$ (8)

$$Z_{\text{2}}=\;Z_{h}\frac{Z_{\text{1}}+Z_{h}tanh\left(\gamma h\right)}{Z_{h}+Z_{\text{1}}tanh\left(\gamma h\right)}$$ (9)

$$Z_{\text{1}}=\;jZ_{o}tan\beta h$$ (10)

where $Z_{h}$ and $Z_{o}$ correspond to the characteristic impedances of the FR4 substrate and air gap, respectively. The propagation constant (β) and phase constant (γ) define the electromagnetic wave behavior in the air layer. The optimized values for C and L are optimized using the Keysight Advanced Design System (ADS) simulator, while the values of the resistors R1 and R2 are kept at 400 Ω.

**Fig 6:**
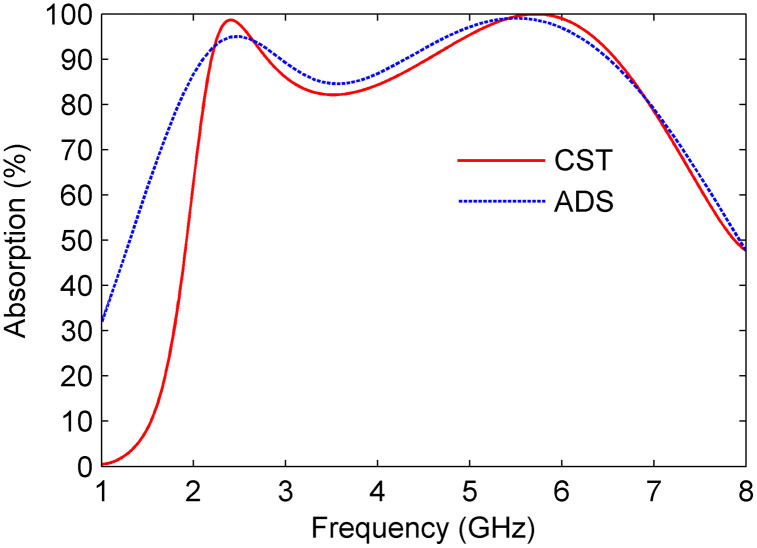
Comparison of simulated absorptivity results obtained from CST and ADS.

Fig 6 shows the Absorption coefficients obtained from CST and ADS simulations, demonstrating a strong correlation between the results of both methods.

**Fig 7:**
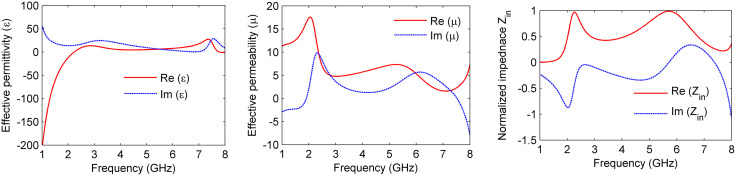
MS properties (a) permittivity (ε), (b) permeability (μ), and (c) normalized impedance $\left(z_{in}\right)$.


To comprehensively evaluate the performance of the proposed design, the fundamental electromagnetic properties, particularly the effective ε and μ, are analyzed in conjunction with the normalized input impedance $\left(Z_{in}\right)$. Fig 7(a) and (b) illustrate the frequency variations of ε and μ along with their real and imaginary components. It is evident from the figures that the real parts of both ε and μ approach zero within the operating frequency range, indicating minimal reflectance. Additionally, the imaginary parts exhibit non-negligible magnitudes, signifying dissipative losses. These losses are primarily responsible for enabling effective absorption within the target frequency spectrum.

$$Z_{in}=\sqrt{\frac{(1+S_{11}{)}^{\text{2}}-(S_{21}{)}^{\text{2}}}{(1-S_{11}{)}^{\text{2}}-(S_{21}{)}^{\text{2}}}}$$ (11)

Fig 7(c) depicts the normalized input impedance derived from the S-parameters using equation (11). At the operating frequencies, the real part of the impedance approaches unity, while the imaginary part approaches zero, indicating an excellent match with the free-space impedance. This impedance matching condition ensures near-unity absorptivity and significantly suppresses reflectance, as the structure effectively minimizes wave reflections.

**Fig 8:**
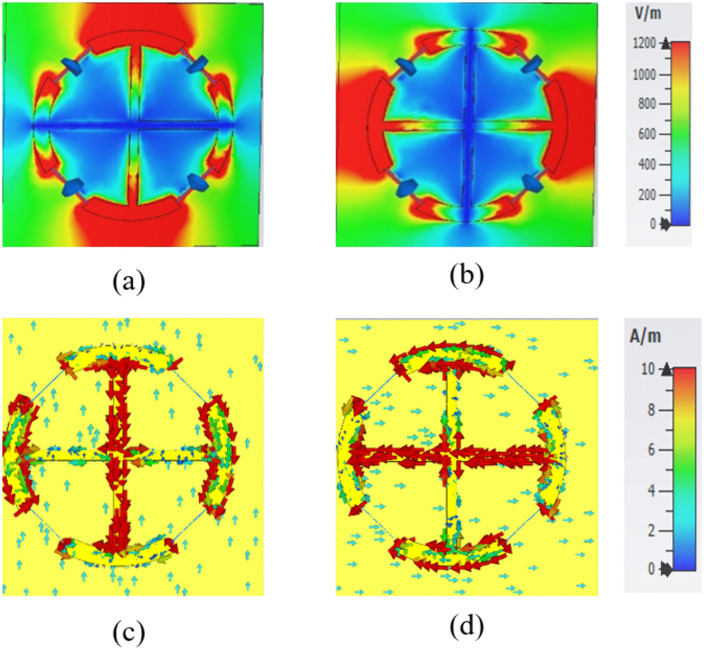
Simulated electromagnetic responses: (a, b) E-field corresponding to TE and TM polarizations, respectively; and (c, d) surface current corresponding to TE and TM polarizations, respectively, at 2.4 GHz.

To better understand the physical mechanism behind the proposed MS absorber, the electric field (E-field) and surface current distributions were analyzed for both TE and TM polarization modes at 2.4 GHz and 5.8 GHz. Fig 8 illustrates the E-field and surface current distributions for both polarizations at 2.4 GHz. The E-field is primarily concentrated along the circular resonator, more prominently along the y-axis for TE polarization and the x-axis for TM polarization, as shown in Fig 8(a) and (b), respectively. The surface current exhibits anti-parallel flow distributions for both modes. For TE polarization, the current is mainly concentrated along the y-oriented arm of the plus-shaped strip, while for TM polarization, it is localized along the x-oriented arm, as depicted in Fig 8(c) and (d).

**Fig 9:**
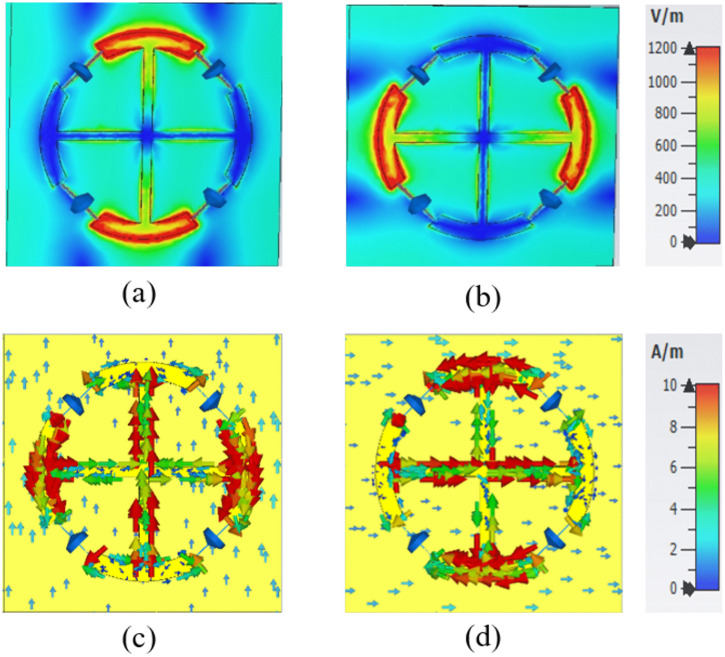
Simulated electromagnetic responses: (a, b) E-field distributions corresponding to TE and TM polarizations, respectively; and (c, d) surface current distributions for TE and TM polarizations, respectively, at 5.8 GHz.

Similarly, Fig 9 presents the E-field and surface current distributions at 5.8 GHz for both TE and TM polarizations. The E-field is concentrated within the circular resonator, aligning along the y-axis for TE polarization and the x-axis for TM polarization, as illustrated in Fig 9(a) and (b), respectively. The surface current distribution exhibits opposing orientations for the two polarization modes. For TE polarization, the current intensity is amplified along the x-axis of the circular resonator, whereas for TM polarization, it is predominantly aligned along the y-axis, as shown in Fig 9(c) and (d). Notably, the resonator exhibits a concentrated current along its peripheral boundaries, with alignment along the x-axis for TE polarization and the y-axis for TM polarization. This enhances the polarization-dependent resonant behavior.

**Fig 10:**
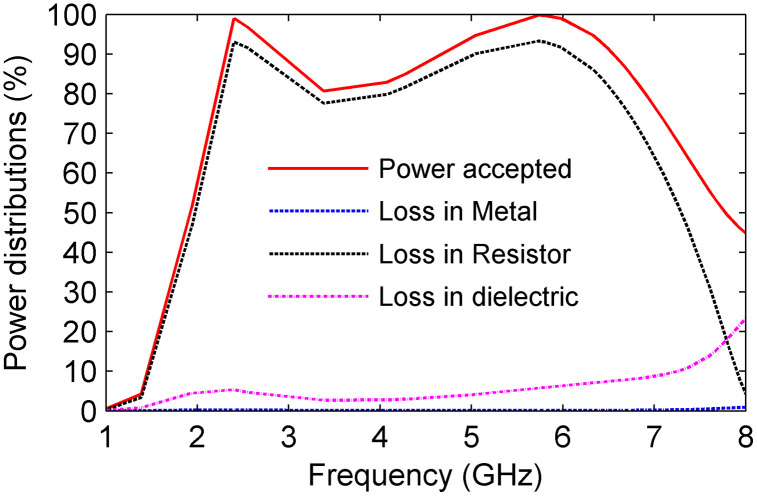
Power distribution into the cell.

To evaluate the absorption efficiency and energy dissipation mechanism of the proposed metasurface absorber, the power distribution within the unit cell was analyzed using CST Microwave Studio. Fig 10 shows the accepted power by the cell and the corresponding losses in the metal, dielectric substrate, and lumped resistors under normal incidence. Since the unit cell is symmetrical and polarization-insensitive, only the TE-mode results are presented. As shown in Fig 10, over 80% of the absorbed power is dissipated in the lumped resistors across the frequency range of 2.1–6.6 GHz, with peak power losses exceeding 93% at 2.4 GHz and 5.8 GHz. The losses in the metal and dielectric layers remain negligible, confirming that the lumped resistors are the dominant loss elements responsible for achieving near-unity absorption.

### 3.3. Absorptivity variation with polarization and incident angles

**Fig 11:**
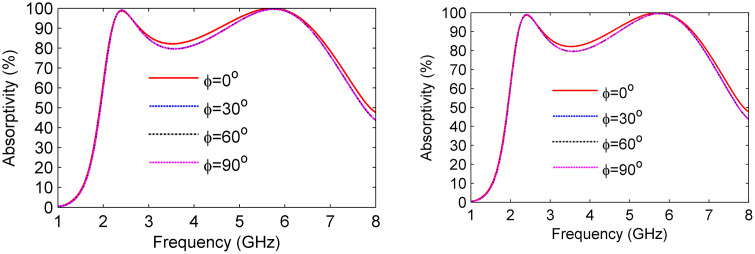
Absorptivity ratio at varying polarization angles (a) TE polarization and (b) TM polarization.

The performance of the proposed broadband MS absorber was comprehensively evaluated by analyzing its absorptivity under various polarization conditions, as illustrated in Fig 11.

In this analysis, the direction of the incident wave was held constant, while the polarization angle was varied, resulting in a rotation of both the E-field and H-field directions. Fig 11 illustrates that the absorptivity response remains stable as the polarization angle (ϕ) shifts from 0° to 90° for both TE and TM polarizations. Due to the symmetric plus-shaped geometry of the resonator, the proposed MS absorber exhibits polarization-independent performance, maintaining nearly identical absorption characteristics for both TE- and TM-polarized incident waves.

**Fig 12:**
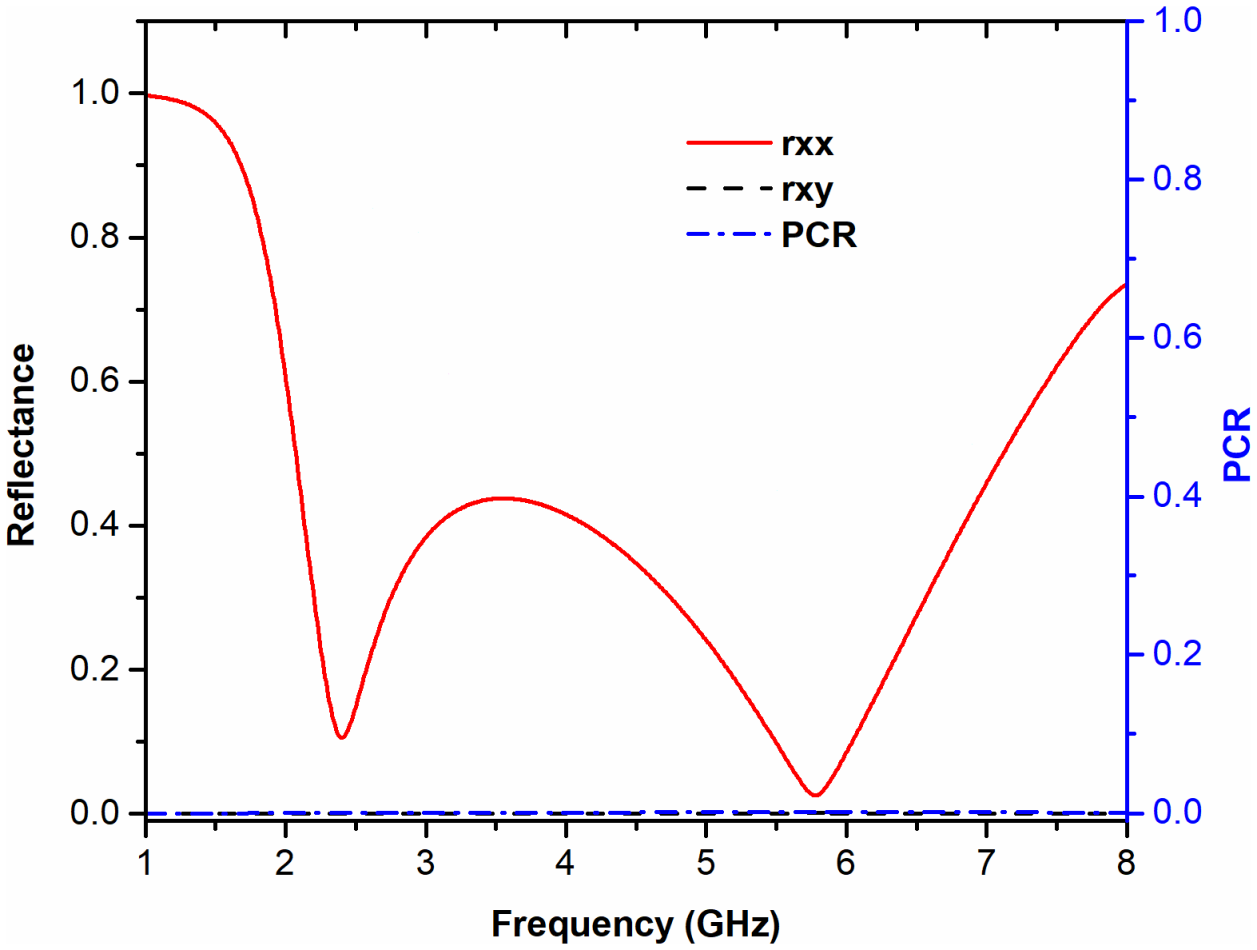
Co-polarized and cross-polarized reflection responses, along with the PCR, under an x-polarized incident wave.

To evaluate the operational performance of the MS structure, particularly its absorption and polarization conversion characteristics, the polarization conversion ratio (PCR) is investigated. The PCR is defined as follows [41]


$$PCR=\frac{|R_{yx}{|}^{\text{2}}}{|R_{yx}{|}^{\text{2}}+|R_{xx}{|}^{\text{2}}}=\frac{|R_{xy}{|}^{\text{2}}}{|R_{xy}{|}^{\text{2}}+|R_{yy}{|}^{\text{2}}}$$ (12)

where $R_{yx}=E_{yx}/E_{xi}$, $R_{xx}=E_{xr}/E_{xi}$ represent cross- and co-polarized reflection coefficients under x-polarized incidence, and $R_{xy}=E_{xr}/E_{yi}$, $R_{yy}=E_{yr}/E_{yi}$ correspond to cross- and co-polarized reflections for y-polarized incidence. The subscripts “i” and “r” represent the incident and reflected waves, respectively. Fig 12 illustrates the co-polarized and cross-polarized reflectance along with the PCR. The PCR consistently approaches zero across the frequency band, confirming that energy dissipation (absorption) dominates over polarization conversion.

**Fig 13:**
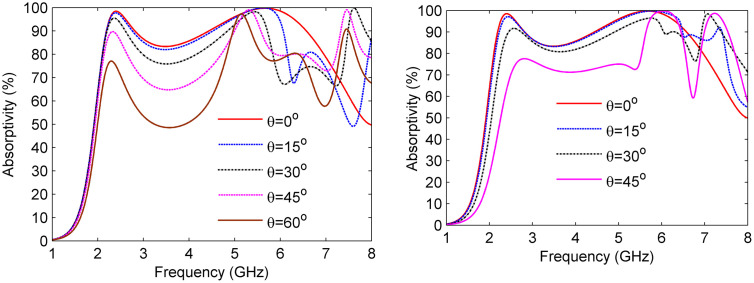
Absorptivity at various oblique incidence angles (a) TE polarization and (b) TM polarization.

To investigate angular stability, the absorptivity of the MS absorber was examined across a range of incidence angles, as illustrated in Fig 13. At TE polarization, where the electric field orientation is fixed, absorptivity decreases with increasing angle but retains high efficiency, exceeding 74% at 2.4 GHz and reaching 96% at 5 GHz, even at extreme angles (θ → 60°). In the case of TM polarization, the resonant frequencies exhibit a slight upward shift, with absorption efficiencies achieving 77% at 2.7 GHz and 99% at 5.9 GHz, under a 45° incidence angle. Furthermore, parasitic resonances at higher angles introduce additional absorption bands, thus broadening the operational spectrum. These results confirm the design’s robustness under various oblique incidence angles while sustaining strong broadband absorption performance.

### 3.4. Parametric study

1) ***The Effect of Loss and Loss-Free Substrate***

**Fig 14:**
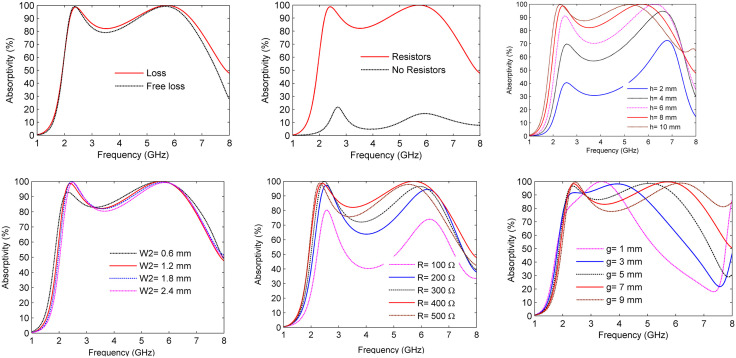
Absorptivity of the MS absorber at several design parameters: (a) absorptivity for loss and free loss substrate, (b) absorptivity for load and unloaded lumped resistors, (c) effect of the air space height (h), (d) effect of various plus resonator’s width ($W_{\text{2}}$), (e) effect of varying lumped resistor loads and split length (g).

To examine the impact of substrate loss on absorption, a full-wave simulation was performed under both lossy and loss-free substrate conditions, as illustrated in Fig 14(a). The observed near-unity absorption confirms that electromagnetic absorption primarily results from the ohmic loss in lumped resistors rather than the dielectric loss in the proposed absorber’s structure.

2) ***The Effect of Loaded/Unloaded Lumped Resistors***

The performance of the MS absorber was evaluated for two configurations: a resistor-integrated design and a resistor-free design, as depicted in Fig 14(b). The resistor-free configuration achieves a modest absorption peak of approximately 20%. In contrast, adding lumped resistors significantly enhances the performance, bringing absorption levels close to unity and expanding the bandwidth. These results highlight the crucial role that resistive elements play in optimizing both the absorptivity and the spectral coverage.

3) ***The Effect of Changing the Air Gap’s Height (h)***

The effect of the air gap’s height (h) on absorptivity was examined by varying *h* from 2 mm to 10 mm. The results indicate that as *h* increases, absorptivity also increases, while the absorption resonances shift to lower frequencies. This shift is attributed to the reduced capacitive coupling between layers. An air gap of *h = 8 mm* was identified as optimal, resulting in near-perfect absorption (~99%) at 2.4 GHz and 5.8 GHz, as depicted in Fig 14(c).

4) ***The Effect of The Plus Resonator’s Width (***$W_{\text{2}}$**)**

The impact of the plus resonator’s width ($W_{\text{2}}$) on the absorptivity of the proposed MS absorber has also been investigated by varying $W_{\text{2}}$ from 0.6 mm to 2.6 mm in 0.6 mm increments, as shown in Fig 14(d). The width of 1.2 mm was selected as optimal, as it achieved near-unity absorptivity at both 2.4 GHz and 5.8 GHz.

5) ***The Effect of Resistor Values***

The influence of lumped resistors integrated into the resonator splits was analyzed for values ranging from 100 Ω to 500 Ω, as shown in Fig 14(e). The absorptivity increased with resistance, peaking at 400 Ω with near-unity absorption of over 99%. This optimal value ensures improved impedance matching with free space, thereby maximizing electromagnetic energy dissipation and minimizing reflection losses.

6) ***The Effect of the Split’s Length (g)***

The absorptivity of the MS absorber was evaluated by systematically varying the split length (g) from 1 mm to 9 mm, as illustrated in Fig 14(f). The results indicate that absorption in the 5 GHz band improves with increasing split lengths. The optimal split length of 7 mm was identified, achieving near-unity absorption of over 99% and a broader operational bandwidth.

## 4. Measurement verification

**Fig 15:**
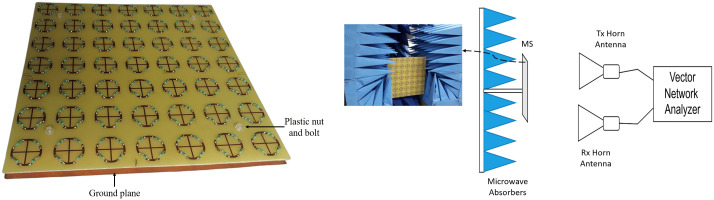
(a) Prototype of the fabricated MS absorber and (b) Schematic representation of the experimental measurement configuration.

To verify the efficacy of the proposed MS absorber, a prototype was fabricated using standard PCB manufacturing techniques on a thick FR4 substrate. The structure consists of a 7 × 7 periodic array of unit cells, with dimensions identical to those used in the simulation. Lumped resistors were integrated into the resonator splits through precision soldering to ensure consistent electrical performance. The fabricated prototype occupies a total area of 245 × 245 mm². A uniform air gap between the substrate and the ground plane was maintained using plastic nuts and bolts, as illustrated in Fig 15(a). According to CST simulations, the dielectric materials of the plastic nuts and bolts have a negligible effect on the reflection coefficients, confirming that the supporting components do not influence the electromagnetic performance of the absorber.

The reflection coefficient measurements were conducted in a free-space configuration using two horn antennas (type HF906) with an Agilent E5071C vector network analyzer, where one antenna served as the transmitter and the other as the receiver. The transmitting antenna radiated the incident electromagnetic wave toward the metasurface prototype, and the receiving antenna captured the reflected signal for analysis. The fabricated absorber was positioned 1m away from the antennas to achieve maximum radiation power, as illustrated in Fig 15(b). The measurements were carried out in an open laboratory environment, and unwanted reflections from nearby objects were minimized by surrounding the setup with microwave absorbers. A copper sheet of identical dimensions was used as a reference reflector for calibration, and the reflected power from the absorber was normalized with respect to that of the copper plate. Multiple measurements were averaged to suppress random noise and improve accuracy. The absorptivity of the prototype was then calculated using equation (3).

**Fig 16:**
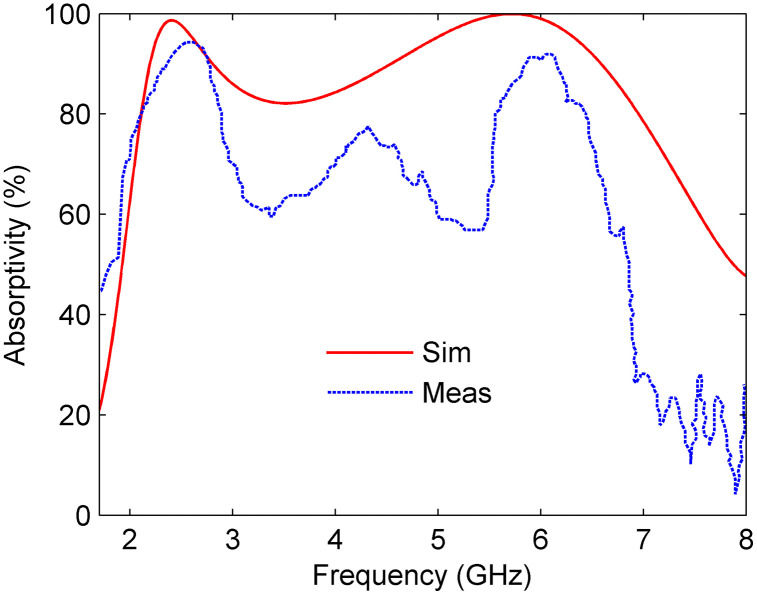
Simulated and measured absorptivity under normal incidence.

A comparison of simulated and measured absorptivity under normal incidence is shown in Fig 16. Both results exhibit two distinct absorption peaks corresponding to the designed resonant modes, with measured absorption exceeding 90% at 2.55 GHz and 5.88 GHz, closely matching the simulated predictions. Minor discrepancies, such as slightly narrower bandwidth and reduced absorptivity in measurements, can be attributed to fabrication tolerances, variations in resistor placement and soldering, and small inconsistencies in the air gap between the substrate and ground plane. Additional measurement-related uncertainties, including residual reflections from the open-space setup, the finite beamwidth of horn antennas, and slight alignment errors, may also contribute. Despite these variations, the overall absorption trends and resonance positions show strong agreement between simulation and experiment, validating the accuracy of the proposed design. The thermal effects in the lumped resistors were not analyzed in this study, as the absorber operates at low ISM-band power levels where self-heating is negligible. However, future work will include electro-thermal analysis and extended measurements under higher power and varying environments to evaluate performance stability and angular robustness.

Table 1 presents a comparative summary of the key performance metrics of the proposed MS absorber against existing designs reported in the literature. It is noted that the proposed MS absorber achieves a higher fractional bandwidth across the frequency range of 2.1–6.96 GHz, which significantly outperforms most prior works. The design maintains a compact overall dimension and a minimal relative thickness, demonstrating stable performance at oblique incidence angles of up to 60°. In contrast to other absorbers that require a greater number of resistive elements [32,39–41] or thicker substrates [35,40], the proposed structure achieves broadband absorption with only four resistive components and a simplified geometry. This results in reduced complexity and cost in the fabrication process. Furthermore, the proposed design exhibits a broad and angularly stable response, underscoring its advantages in practical applications, including wireless communication, radar cross-section reduction, and electromagnetic interference (EMI) suppression. Overall, the findings demonstrate that the proposed MS absorber achieves an optimal balance of bandwidth, compactness, and design simplicity, surpassing the performance of most existing alternatives in terms of both efficiency and practicality.

**Table 1:** Comparative analysis of the MS absorber with literature approaches.

Ref.	Frequency range	Size	Relative Thickness	Max. Oblique incident	No. Resistor	Fractional Bandwidth
[32]	2.85-5.31 GHz	0.29λ_2.85_	0.063λ_2.85_	45^o^	8	60.3%
[34]	860-960 MHz	0.6λ_0.86_	0.06λ_0.86_	30^o^	4	16%
[35]	1.94-2.99 GHz	0.36λ_1.94_	0.12λ_1.94_	45^o^	4	42.6%
[38]	2.11-3.89 GHz	0.14λ_2.11_	0.1λ_2.11_	70^o^	4	59.3%
[39]	5.44-10.21 GHz	0.27λ_5.44_	0.33λ_5.44_	75^o^	4	61%
[42]	6.76-14.96 GHz	0.31λ_6.76_	0.12λ_6.76_	50^o^	6	75%
[43]	6.7-20.58 GHz	0.3λ_6.7_	0.07λ_6.7_	45^o^	8	101.8%
**Pro**	**2.1-6.96 GHz**	**0.24λ**_**2.1**_	**0.06λ**_**2.1**_	**60** ^**o**^	**4**	**107.3%**

## 5. Conclusion

This study introduces the design, simulation, and experimental validation of a compact, polarization-insensitive, broadband MS absorber specifically engineered to operate at the ISM frequencies of 2.4 GHz and 5.8 GHz. The proposed architecture features a plus-shaped split-ring resonator (SRR), integrated with four lumped resistors, which is patterned on an FR4 substrate and supported by a copper ground plane. An 8 mm air gap has been introduced to enhance impedance matching and improve bandwidth performance. Full-wave simulations demonstrated near-unity absorption efficiencies exceeding 98% at the target frequencies, while experimental results achieved absorption levels above 90%, showing strong agreement with simulated predictions. The absorber also exhibits a wide fractional bandwidth of over 107% across the frequency range of 2.1 to 6.96 GHz, maintaining stable performance for both TE and TM polarizations under oblique incidence angles of up to 60°. Electromagnetic field and surface current analyses reveal that resistive dissipation is the primary absorption mechanism, with negligible contributions from dielectric and conductive losses. Experimental results obtained from a fabricated prototype align closely with simulation data, confirming the design’s accuracy. Compared to previous works, the proposed absorber offers enhanced frequency selectivity, reduced structural complexity, and a lower-profile configuration, positioning it as a promising candidate for practical applications in wireless communication systems, electromagnetic interference (EMI) mitigation, and stealth technology.

## Supporting information

S1 Data (RAR)
